# Coccidioidomycosis as a Potential Trigger for Sarcoidosis: A Case Study

**DOI:** 10.7759/cureus.53272

**Published:** 2024-01-31

**Authors:** Poonam Tawde, Salim Mohammad

**Affiliations:** 1 Physiotherapy, Sancheti institute for Orthopaedics and Rehabilitation, Pune, IND; 2 Medical School, Avalon University School of Medicine, Willemstad, CUW; 3 Critical Care, Chandler Regional Medical Center, Chandler, USA

**Keywords:** diagnostic challenges, interdisciplinary collaboration, chronic kidney disease, hypercalcemia, sarcoidosis, valley fever, coccidioidomycosis

## Abstract

This study explores the complex interplay between coccidioidomycosis (valley fever) and sarcoidosis through a detailed case study of a 54-year-old male patient. The patient presented with elevated calcium levels, chronic kidney disease (CKD), and unintended weight loss. Interdisciplinary collaboration between nephrologists and pulmonologists played a crucial role in navigating the intricate medical challenges, including hypercalcemia, renal dysfunction, and pulmonary anomalies. The diagnostic journey involved extensive laboratory findings uncovering the involvement of both infectious agents and granulomatous disorders. The patient exhibited positive cocci IgG antibodies, indicating coccidioidomycosis. Further complications included glomerulonephritis, as revealed by ongoing systemic inflammation. Tailored management strategies were implemented, including corticosteroid therapy for sarcoidosis-related inflammation and antifungal interventions for coccidioidomycosis. Vigilant monitoring of renal function, hypercalcemia, and weight loss was essential for comprehensive patient care. The study underscores the significance of interdisciplinary collaboration, systematic diagnostics, and personalized patient care in managing complex medical presentations and contributes to understanding the interplay between these two conditions.

## Introduction

Coccidioidomycosis, commonly known as valley fever, is a fungal infection caused by the inhalation of spores of the dimorphic fungi *Coccidioides immitis* or *Coccidioides posadasii* [1]. This infection primarily affects the respiratory system and is endemic to arid regions, including the southwestern United States [1]. On the other hand, sarcoidosis is a systemic granulomatous disorder of unclear etiology, characterized by the formation of non-caseating granulomas in various organs, most frequently the lungs [2]. Despite their distinct etiologies, a complex interplay has been suggested between coccidioidomycosis and sarcoidosis, wherein *Coccidioides* infection might trigger or exacerbate sarcoidosis in susceptible individuals. The etiology of coccidioidomycosis involves the inhalation of fungal arthroconidia present in the soil, leading to primary pulmonary infection or dissemination to other organs. The pathogenesis of sarcoidosis remains enigmatic, with proposed hypotheses ranging from infectious agents to genetic predisposition and immune dysfunction [2]. A potential connection between infections and sarcoidosis pathogenesis has been explored, including the role of mycobacteria and *Propionibacterium acnes* [2].

A study conducted by Kuberski and Yourison explored the idea that *Coccidioides* infection could play a role in triggering or worsening sarcoidosis, a systemic inflammatory disorder [3]. The study focused on two concepts: immune dysregulation and molecular mimicry. With regard to immune dysregulation, the researchers suggested that the immune response activated against *Coccidioides* might go awry, leading to excessive or abnormal inflammation. This response could contribute to the formation of granulomas, which are also seen in sarcoidosis. Concerning molecular mimicry, they were of the opinion that shared structures between *Coccidioides* and human tissues might cause the immune system to target both the infection and the body's tissues. This cross-reactivity could lead to inflammation resembling sarcoidosis. The study's findings indicated that certain immune pathways and molecules were commonly elevated in patients with both conditions [3]. According to Valeyre et al. [2] and Kuberski and Yourison [3], the clinical presentation of coccidioidomycosis ranges from mild flu-like symptoms to severe pneumonia, and disseminated cases can involve skin lesions, bone pain, and meningitis. Sarcoidosis presents a broad spectrum of manifestations, commonly involving the lungs with symptoms such as cough, dyspnea, and chest pain. Systemic symptoms like fever, fatigue, and weight loss can also occur. The management of coccidioidomycosis includes antifungal therapy, particularly in severe or disseminated cases. Sarcoidosis management varies based on the extent of organ involvement and symptoms, often involving corticosteroids to suppress inflammation. Complications of coccidioidomycosis encompass chronic lung disease, abscess formation, and dissemination to vital organs. Sarcoidosis complications include progressive fibrosis, organ dysfunction, and secondary infections [3].

In this case report, we present a rare co-occurrence of coccidioidomycosis and sarcoidosis in a patient, highlighting the complex interplay between these two conditions and the challenges in their diagnosis and management.

## Case presentation

In this case study, we delve into the complex medical journey of a 54-year-old male patient, navigating a maze of clinical challenges that encompass hypercalcemia, pulmonary anomalies, and chronic kidney disease (CKD). This intricate case highlights the interplay of various medical conditions and emphasizes the critical role of a systematic and interdisciplinary approach in both diagnosis and management.

The patient's admission was prompted by an alarming discovery during routine laboratory investigations: notably elevated calcium levels. A comprehensive review of his medical history revealed a backdrop of stage IV CKD and a history of hypertension. These underlying health concerns immediately cast a spotlight on the intricate nature of the case. Adding to the complexity, the patient had experienced a dramatic and unintended weight loss of 30-50 pounds within a remarkably short timeframe. Upon admission, the patient's demeanor was notably composed, appearing alert and generally well. A thorough physical examination did not immediately reveal any acute distress or overt symptoms. However, a closer examination of the laboratory results painted a more nuanced picture. The presence of hypercalcemia, with a calcium level measuring 14.8, was conspicuous. This was accompanied by elevated creatinine levels (4.00) and hyperglycemia (glucose level: 149), adding layers of intricacy to the patient's clinical profile. Importantly, anemia, characterized by a hemoglobin level of 12.8, and hyponatremia (sodium level: 133) further deepened the diagnostic challenge.

**Figure 1:**
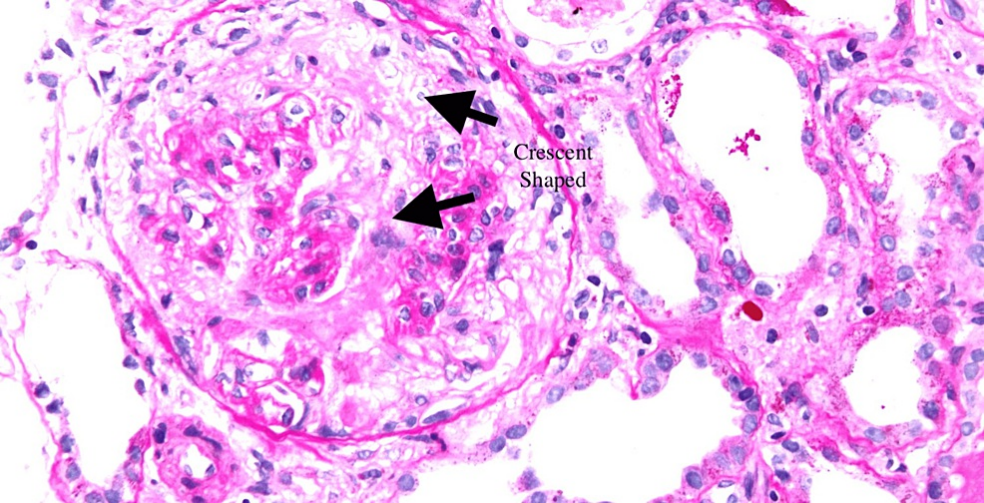
The glomerulus displays a crescent-shaped zone characterized by hypercellularity and accompanied by an inflammatory infiltrate (macrophages indicated by arrow).

Given the multifaceted presentation and the intricacy of the clinical challenges, a collaborative and multidisciplinary diagnostic approach was deemed essential. Experts in nephrology and pulmonology were enlisted to unravel the intricate web of medical complexities that this case presented. Nephrologists were consulted to ascertain the feasibility of a kidney biopsy, while pulmonologists embarked on a thorough investigation to uncover the underlying cause of the observed cavitary lung lesion. The patient's complex presentation prompted a meticulous examination of laboratory findings. The detection of positive cocci IgG antibodies pointed towards the involvement of an infectious agent. However, a bone marrow biopsy provided some relief by ruling out malignancy as an underlying cause. Notably, the renal biopsy introduced an entirely new dimension to the case, with findings suggestive of glomerulonephritis (Figure 1).

**Figure 2:**
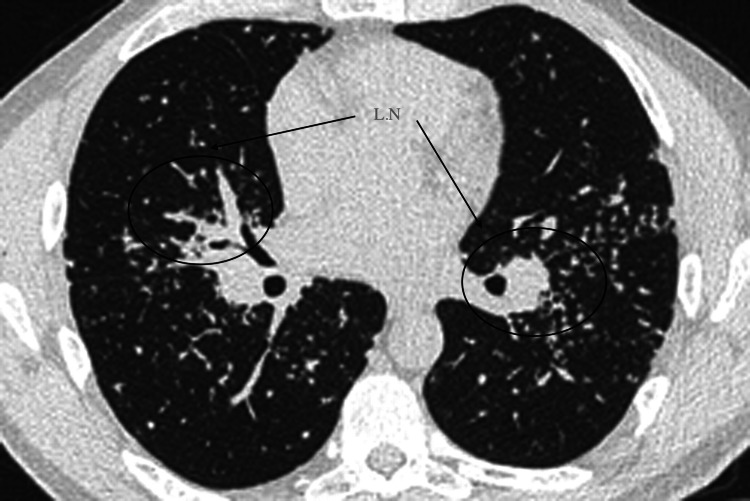
The CT scan of the chest revealed hilar lymphadenopathy, indicating the enlargement of lymph nodes in the pulmonary hilum. Lymph nodes were observed in the right upper lobe, interlobar region, middle lobe, and lower lobe, as well as in the left upper lobe, interlobar region, and lower lobe.

Throughout this diagnostic process, consistently elevated levels of angiotensin-converting enzyme (ACE) and persistent hypercalcemia continued to underscore the presence of ongoing systemic inflammation. Bronchoscopy emerged as a crucial diagnostic tool in the pursuit of understanding the patient's pulmonary health. While the examination of the airways revealed normal findings, the discovery of bilateral hilar lymphadenopathy raised questions regarding the potential extent of lung involvement (Figure 2). In an attempt to shed light on the origin of the cavitary lung lesion, multiple needle biopsies were meticulously conducted on both right and left hilar lymph nodes. They identified a typical pattern of noncaseating granulomas without necrosis, with clustered, well-demarcated lymph nodes. Right and left paratracheal lymph nodes and subcarinal lymph nodes were visible.

The intricacies of the patient's clinical scenario demanded a holistic and multifaceted management strategy. The convergence of factors, including cocci infection, cavitary lung lesion, sarcoidosis, CKD, and hypercalcemia, necessitated tailored approaches to address each facet of the patient's condition. Corticosteroid therapy emerged as a promising avenue to tackle sarcoidosis, while antifungal interventions were initiated to combat the cocci infection. The management of this case extended beyond mere medical interventions. It encompassed vigilant monitoring and personalized patient care. Given the complexity of the patient's renal function, continual assessment was imperative. Additionally, the complications stemming from hypercalcemia necessitated meticulous oversight, and the patient's significant weight loss prompted recommendations for dietary supplementation once his oral intake returned to a more normal course. 

## Discussion

The presented case study illustrates the intricate interplay of multiple medical conditions, including hypercalcemia, pulmonary anomalies, and CKD. The diagnostic process and management strategies employed highlight the complexity of modern medical practice and the importance of a comprehensive and interdisciplinary approach. The case's complexity was evident from the patient's medical history, which included stage IV CKD and hypertension [4].^ ^Such underlying conditions can significantly influence diagnostic and therapeutic decisions. The dramatic weight loss experienced by the patient further complicated the diagnostic process, as unintended weight loss can be associated with various underlying systemic conditions [4]. 

Laboratory findings played a pivotal role in unraveling the case's intricacies. The identification of hypercalcemia prompted consideration of various etiologies, including hyperparathyroidism, malignancy, and granulomatous disorders [5]. The elevated creatinine levels and hyperglycemia were indicative of the patient's underlying CKD and possible metabolic disturbances [6]. Additionally, anemia and hyponatremia further highlighted the multifaceted nature of the patient's clinical presentation. The collaborative diagnostic approach involving nephrology and pulmonology specialists proved crucial in navigating the diagnostic challenges. Such interdisciplinary collaboration is essential in complex cases where the involvement of multiple organ systems is apparent [7]. The nephrology team's consideration of a kidney biopsy was integral in determining the underlying cause of the patient's renal dysfunction, as kidney biopsies can provide valuable insights into the nature of glomerular diseases [8]. Simultaneously, the pulmonology team's investigation into the cavitary lung lesion demonstrated the significance of bronchoscopy in evaluating pulmonary health.

The identification of positive cocci IgG antibodies raised the possibility of an infectious etiology. Such findings underscore the importance of considering infectious agents in the differential diagnosis of granulomatous diseases [9]. The negative bone marrow biopsy ruling out malignancy alleviated concerns of a hematological disorder contributing to the patient's presentation. Notably, the renal biopsy's revelation of glomerulonephritis introduced a new dimension to the case, emphasizing the complexity of renal involvement in systemic diseases [10]. Consistently elevated ACE levels and persistent hypercalcemia pointed towards ongoing systemic inflammation, potentially related to the patient's sarcoidosis and CKD. The investigation of the pulmonary component was equally enlightening. The absence of acute pulmonary issues in bronchoscopy findings was reassuring, ruling out acute respiratory pathology. The presence of bilateral hilar lymphadenopathy, however, suggested broader lung involvement and necessitated further investigation.

Multiple needle biopsies were instrumental in shedding light on the origin of the cavitary lung lesion, underscoring the value of targeted tissue sampling in complex cases. Additionally, the patient's weight loss prompted dietary supplementation recommendations, highlighting the importance of addressing nutritional aspects to optimize patient outcomes [10]. The management of this intricate case exemplified the need for a holistic and multidisciplinary approach and tailored treatment.

## Conclusions

This case study of a 54-year-old male patient with hypercalcemia, pulmonary anomalies, and CKD highlights the intricate nature of medical challenges. The systematic and interdisciplinary approach employed in diagnosis and management proved crucial in unraveling the complexities. The convergence of factors, including cocci infection, sarcoidosis, CKD, and hypercalcemia, demanded a holistic and tailored management strategy. The collaborative efforts of specialists, meticulous examinations, and tailored management strategies reflect the complexity of contemporary medicine in deciphering health complexities. The case underscores the importance of a comprehensive, customized, and coordinated approach in navigating the intricate landscape of health complexities. It serves as a compelling testament to the dynamic and ever-evolving nature of modern medicine, which continually adapts to decipher the intricacies of health and illness.
